# SIRT2 inhibits non-small cell lung cancer cell growth through impairing Skp2-mediated p27 degradation

**DOI:** 10.18632/oncotarget.7816

**Published:** 2016-03-01

**Authors:** Ziming Li, Jia Huang, Hong Yuan, Zhiwei Chen, Qingquan Luo, Shun Lu

**Affiliations:** ^1^ Shanghai Lung Tumor Clinical Medical Center, Shanghai Chest Hospital, Shanghai Jiao Tong University, Shanghai 200030, People's Republic of China

**Keywords:** lung cancer, cell cycle, acetylation

## Abstract

Skp2 is a component of the E3 ubiquitin ligase which promotes the ubiquitination-associated degradation of a cyclin-dependent kinase inhibitor, p27, resulting in increases in non-small cell lung cancer (NSCLC) cell growth. We recently showed that down-regulation of Sirtuin deacetylases 2 (SIRT2) in NSCLC increased cancer cell growth through suppressing p27. However, the underlying mechanisms remain unknown. Here, we examined the relationship between SIRT2 and Skp2 in regulation of NSCLC cell growth through p27. We found that the levels of SIRT2 significantly decreased, while the levels of Skp2 significantly increased in NSCLC specimens, compared to the paired non-tumor lung tissue. The levels of SIRT2 and Skp2 inversely correlated. Low SIRT2 levels were associated with poor patients' survival. Moreover, in several lung cancer cell lines, the SIRT2 levels significantly decreased and the Skp2 levels significantly increased. Overexpression of SIRT2 promoted Skp2 deacetylation and degradation, resulting in increases in p27 and suppression of NSCLC cell growth, whereas knockdown of Skp2 inhibited Skp2 deacetylation and degradation, resulting in decreases in p27 and increases in NSCLC cell growth. The deacetylation of Skp2 by SIRT2 and degradation of p27 by Skp2 were significantly inhibited by histone deacetylase inhibitor and proteasome inhibitor, respectively. Finally, SIRT2 and Skp2 co-immunoprecipitated in NSCLC cells. Together, our data suggest that SIRT2 may induce Skp2 deacetylation and subsequent degradation to abolish the effects of Skp2 on p27 to affect NSCLC cell growth. Thus, re-expression of SIRT2 may be a promising strategy for treating NSCLC.

## INTRODUCTION

As one of the most common cancer types worldwide and account for a significant number of cancer-associated fatality, Lung cancer is a major threat for public health [1-3]. Non-small cell lung cancer (NSCLC) is the most common Lung cancer, and is often diagnosed at an advanced stage when it displays a poor prognosis, largely resulting from the fast-growing nature of the cancer cells and their early metastases [4]. In the recent years, our knowledge on the molecular mechanisms and biology of NSCLC has been improved, by the introduction of new therapeutic agents and approaches into lung cancer treatment [5-11]. However, the overall 5-year survival rate is still below 4% [12]. Hence, further elucidation of molecular regulation of NSCLC cell growth appears to be critical for improving therapeutic outcome and the overall 5-year survival rate of the patents.

Sirtuins are mammalian homologs of the yeast silent information regulator 2 (SIR2), the histone deacetylases that utilize nicotinamide adenine dinucleotide to adapt their functions [13-15]. In mammals, there are seven homologs of SIR2 (SIRT1-7), of which SIRT1 has been mostly studied and found to play a key role in energy metabolism, telomeric maintenance, and genomic stability by targeting and deacetylating some non-histone proteins [13-15]. Recently, SIRT2 has attracted more attention, since SIRT2 is found to mainly locate in cytoplasm and associated with mitotic apparatus during the cell cycle [13-15]. Moreover, increasing evidence has suggested that SIRT2 is involved in tumorigenesis [16-19]. SIRT2 deficiency causes impairment of cell mitosis, while SIRT2-deficient mice have a higher propensity for developing tumors. Furthermore, SIRT2 expression is down-regulated in some cancers, suggesting that SIRT2 may be a tumor-suppressor [16-19]. We have recently shown that SIRT2 is down-regulated in NSCLC, and overexpression of SIRT2 inhibits growth of NSCLC cells through increasing cellular p27 [20]. However, the underlying mechanisms remains elusive.

Skp2 is a component of the E3 ubiquitin ligase Skp, Cullin, F-box containing complex (SCF) that specifically promotes the ubiquitination-associated degradation of CDK inhibitor p27 [21-23]. Under physiological conditions, Skp2 controls the initiation of mitosis in that its expression peaks at the S and G2 phases, but not G0 and G1 phases [21-23]. The increased expression of Skp2 has been shown in many different types of cancers [24-28], including lung cancer [29-33]. Moreover, a recent study showed that deacetylation of FOXO3 by SIRT1 or SIRT2 facilitated Skp2-mediated FOXO3 poly-ubiquitination and proteasomal degradation [34]. Nevertheless, whether Skp2 may be deacetylated by SIRT2 in lung cancer cells is unknown.

Here, we examined the relationship between SIRT2 and Skp2 in NSCLC. We found that the levels of SIRT2 significantly decreased, while the levels of Skp2 significantly increased in NSCLC specimens, compared to the paired non-tumor lung tissue. The levels of SIRT2 and Skp2 inversely correlated. Low SIRT2 levels were associated with poor patients' survival. Moreover, in several lung cancer cell lines, the SIRT2 levels significantly decreased and the Skp2 levels significantly increased. Overexpression of SIRT2 promoted Skp2 deacetylation and degradation, resulting in increases in p27 and suppression of NSCLC cell growth, whereas knockdown of Skp2 inhibited Skp2 deacetylation and degradation, resulting in decreases in p27 and increases in NSCLC cell growth. The deacetylation of Skp2 by SIRT2 and degradation of p27 by Skp2 were significantly inhibited by histone deacetylase inhibitor and proteasome inhibitor, respectively. Finally, SIRT2 and Skp2 co-immunoprecipitated in NSCLC cells. Together, our data suggest that SIRT2 may induce Skp2 deacetylation and subsequent degradation to abolish the effects of Skp2 on p27 to affect NSCLC cell growth. Thus, re-expression of SIRT2 may be a promising strategy for treating lung cancer.

## RESULTS

### Low SIRT2 is detected in NSCLC, correlates with high Skp2 and poor patient survival

**Figure 1:**
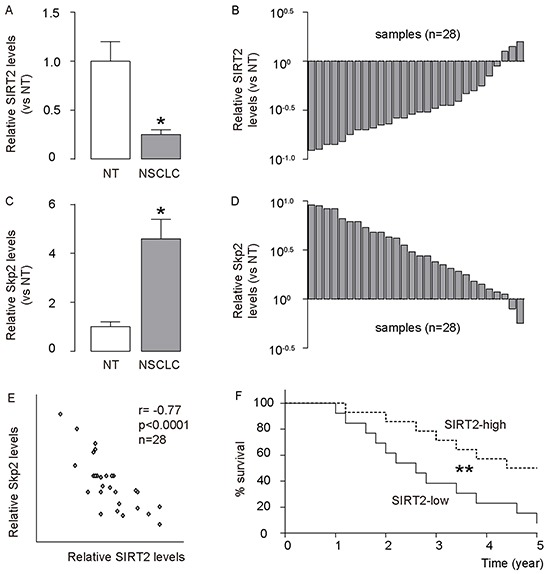
Low SIRT2 is detected in NSCLC, and correlates with high Skp2 and poor patient survival The levels of SIRT2 and Skp2 were analyzed in 28 NSCLC specimens, compared to paired adjacent non-tumor lung tissue (NT). **A–B.** The levels of SIRT2 in NSCLC specimens were analyzed by Western blot, compared to NT, shown by mean±SD (A), and by individual values (B). **C–D.** The levels of Skp2 in NSCLC specimens were analyzed by Western blot, compared to NT, shown by mean±SD (C), and by individual values (D). **E.** A strong inverse correlation was detected between the levels of SIRT2 and Skp2 (r= −0.77; p < 0.0001; n=28). **F.** The 28 patients were followed-up for 5 years. The median value of all 28 cases was chosen as the cutoff point for separating SIRT2-high-expressing cases (n=14) from SIRT2-low-expressing cases (n=14). Kaplan-Meier curves were used to show survival. *p<0.05. **p<0.01.

We analyzed the levels of SIRT2 and Skp2 in age-controlled 28 NSCLC specimens, compared to paired adjacent non-tumor lung tissue (NT). We found that NSCLC specimens contained significantly lower levels of SIRT2, compared to NT, shown by quantification (Figure 1A), and by individual values (Figure 1B). Moreover, we found that NSCLC specimens contained significantly higher protein levels of Skp2, compared to NT, shown by quantification (Figure 1C), and by individual values (Figure 1D). Then, we compared the levels of SIRT2 and Skp2 protein in the same sample. A strong inverse correlation was detected between the levels of SIRT2 and Skp2 (Figure 1E, r= −0.77; p < 0.0001; n=28), suggesting a causal relationship between SIRT2 and Skp2 protein level in NSCLC. Next, we investigated whether the levels of SIRT2 may correlate with overall survival of NSCLC patients. The 28 patients were followed-up for 5 years. The median value of all 28 cases was chosen as the cutoff point for separating SIRT2-high-expressing cases (n=14) from SIRT2-low-expressing cases (n=14). Kaplan-Meier curves indicated that NSCLC patients with low SIRT2 levels had a significantly shorter overall survival (Figure 1F), suggesting that low SIRT2 levels in NSCLC may be associated with poor patient survival.

### Low SIRT2 and high Skp2 are detected in NSCLC cell lines

**Figure 2:**
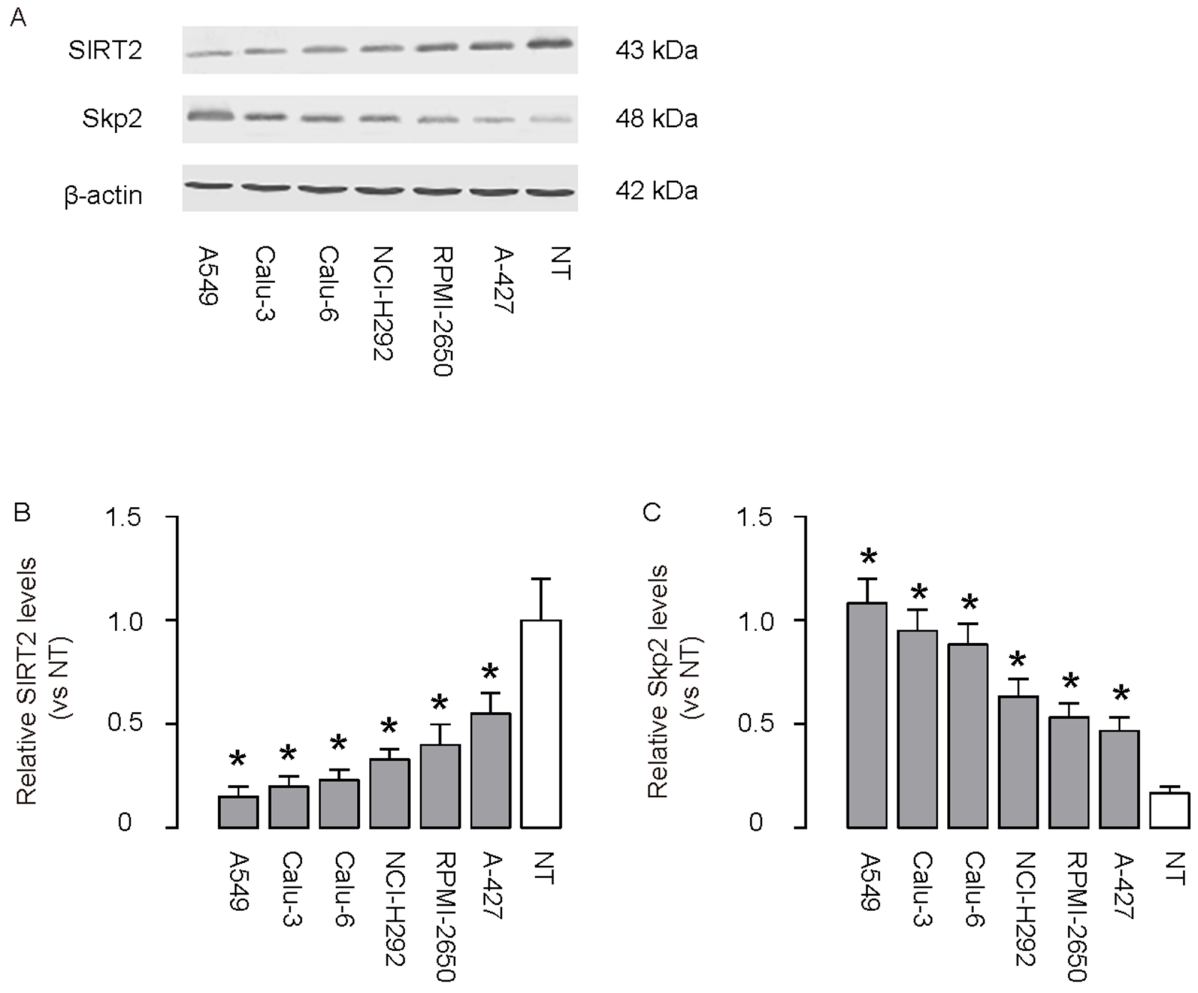
Low SIRT2 and high Skp2 are detected in lung cancer cell lines **A–C.** The levels of SIRT2 and Skp2 were analyzed in 6 commonly used lung cancer cell lines, compared to NT, shown by representative Western blots (A), and by quantification for SIRT2 (B) and for Skp2 (C), and by quantification (Figure 2C). *p<0.05. N=5.

We then analyzed the levels of SIRT2 and Skp2 in 6 commonly used lung cancer cell lines, compared to NT. We found that lung cancer cell lines contained significantly lower levels of SIRT2, compared to NT, shown by representative Western blots (Figure 2A), and by quantification (Figure 2B). Moreover, we found that lung cancer cell lines contained significantly higher levels of Skp2, compared to NT, shown by representative Western blots (Figure 2A), and by quantification (Figure 2C). Thus, our data on lung cancer cell lines are consistent with those from patients' specimens, suggesting that lung cancer cells may have decreased SIRT2 and increased Skp2.

### SIRT2 inhibits Skp2 in NSCLC cells

**Figure 3:**
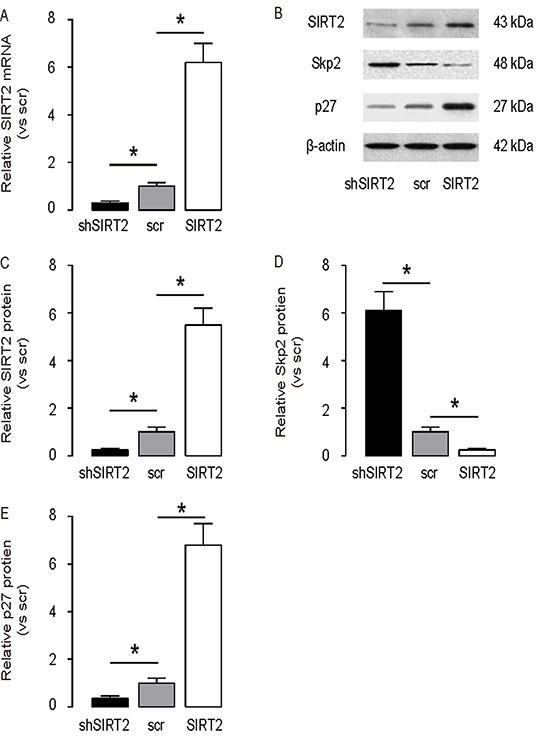
SIRT2 inhibits Skp2 in NSCLC cells SIRT2 levels were modified in A549 cells by transfection the cells with either SIRT2, or shSIRT2 or a scrambled sequence as a control (scr). **A.** RT-qPCR for SIRT2 mRNA in SIRT2-modified A549 cells. **B.** Representative Western blots for SIRT2, Sep2 and p27 in SIRT2-modified A549 cells. **C–E.** Quantification for SIRT2 (C), Sep2 (D) and p27 (E) protein in SIRT2-modified A549 cells. *p<0.05. N=5.

In order to figure out whether SIRT2 may regulate Skp2 in NSCLC cells, we used A549 cells, the most frequently used human NSCLC cell line, to study this question. We modified SIRT2 levels in A549 cells by transfection the cells with either SIRT2, or shSIRT2 or a scrambled sequence as a control (scr). First, we confirmed the modification of the SIRT2 levels in these cells by RT-qPCR (Figure 3A), and by Western blot (Figure 3B-3C). We found that overexpression of SIRT2 in A549 cells significantly decreased the Skp2 levels, by representative blots (Figure 3B), and by quantification (Figure 3D). Moreover, we also examined the levels of p27, a downstream target for Skp2. We found that overexpression of SIRT2 also significantly increased the p27 protein levels, while depletion of SIRT2 significantly decreased the p27 protein levels in A549 cells, by representative blots (Figure 3B), and by quantification (Figure 3E). These data are consistent with previous reports on the effects of SIRT2 and Skp2 on p27. Thus, our data suggest that SIRT2 may inhibit Skp2 to increase p27 in NSCLC cells.

### SIRT2 suppression increases NSCLC cell growth

**Figure 4:**
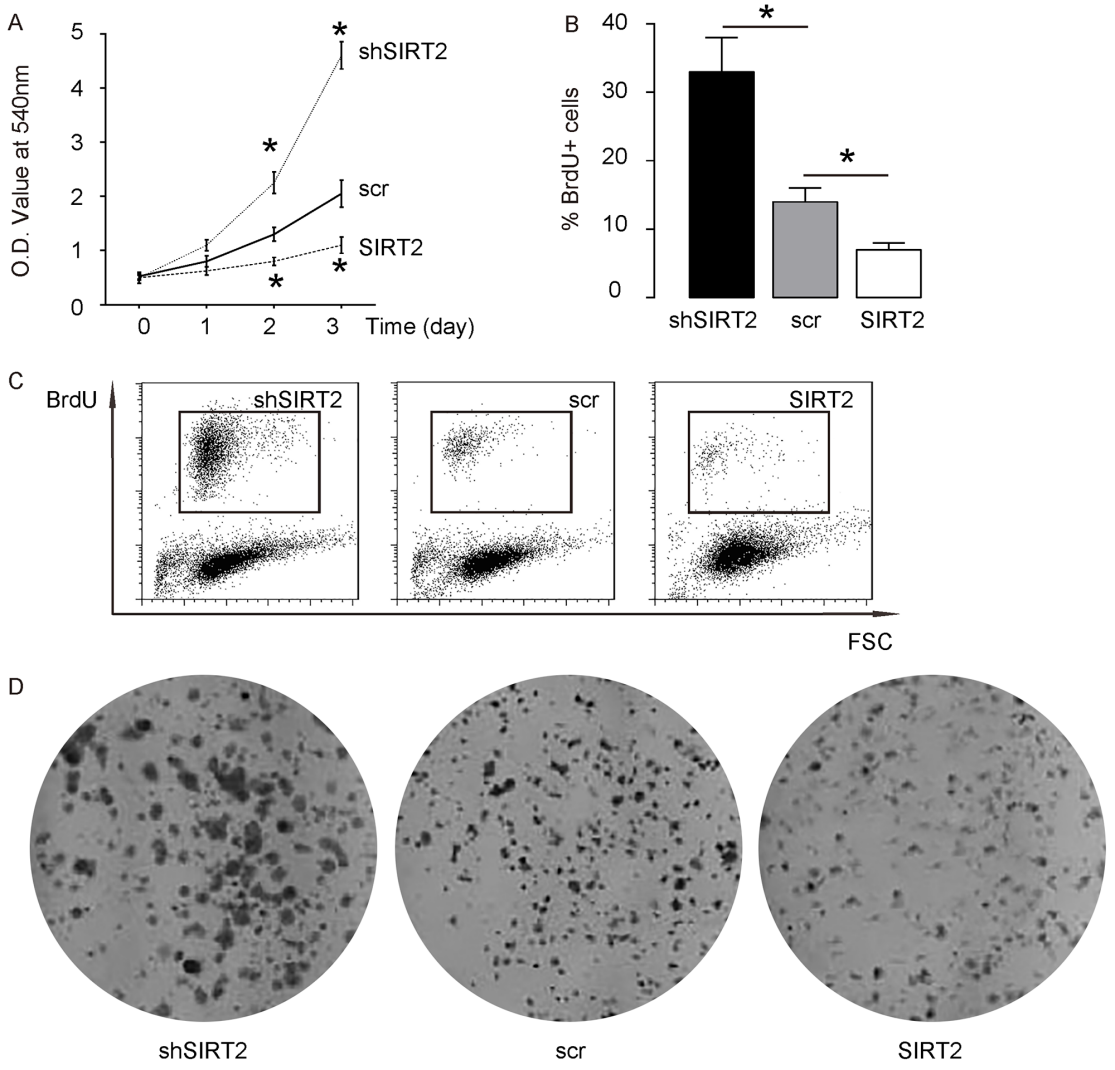
SIRT2 suppression increases NSCLC cell growth **A.** Cell growth of SIRT2-modified A549 cells in an MTT assay. **B–C.** Cell proliferation of SIRT2-modified A549 cells in a proliferation assay, by quantification (B), and by representative flow charts (C). **D.** Tumor colony formation of SIRT2-modified A549 cells. *p<0.05. N=5.

Next, we examined the effects of SIRT2 modification on A549 cell growth. We found that overexpression of SIRT2 significantly decreased cell growth in an MTT assay, while depletion of SIRT2 significantly increased cell growth (Figure 4A). In a proliferation assay, overexpression of SIRT2 significantly decreased BrdU incorporation in A549 cells, while depletion of SIRT2 significantly increased BrdU incorporation in A549 cells, by quantification (Figure 4B), and by representative flow charts (Figure 4C). Moreover, overexpression of SIRT2 seemed to decrease tumor clone formation in A549 cells, while depletion of SIRT2 seemed to increase tumor colony formation in A549 cells (Figure 4D). Together, these data suggest that SIRT2 suppression may increase NSCLC cell growth, possibly through Skp2-mediated p27 degradation.

### SIRT2 regulates p27 levels through Skp2-mediated p27 degradation

**Figure 5:**
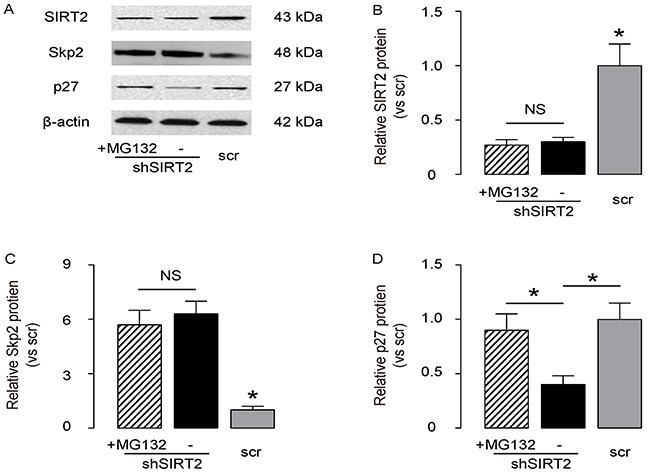
SIRT2 regulates p27 levels through Skp2-mediated p27 degradation The SIRT2-depleted A549 cells were exposed with MG132, a specific proteasome inhibitor to reduce the cellular degradation of ubiquitin-conjugated proteins. **A.** The levels of SIRT2, Skp2 and p27 in SIRT2-depleted A549 cells with/with MG132 were analyzed by Western blot, shown by representative Western blot. **B–D.** Quantification for SIRT2 (B), Sep2 (C) and p27 (D) protein in SIRT2-modified A549 cells. *p<0.05. NS: non-significant. N=5.

Then, we examined how SIRT2 regulated p27 levels in NSCLC cells. Based on literature, Skp2 may induce the ubiquitination-associated degradation of p27. Since we have shown that SIRT2 inhibits Skp2 in NSCLC cells, we aimed to evaluate whether SIRT2 may regulate p27 levels through Skp2-mediated p27 ubiquitination and degradation. We thus exposed SIRT2-depleted A549 cells with MG132, a specific proteasome inhibitor to reduce the cellular degradation of ubiquitin-conjugated proteins. We found that MG132 did not alter the levels of SIRT2 and Skp2 in SIRT2-depleted A549 cells, by representative Western blot (Figure 5A) and by quantification (Figure 5B-5C), but abolished the effects of SIRT2 depletion on the suppression of p27, by representative Western blot (Figure 5A) and by quantification (Figure 5D). These data suggest that SIRT2 indeed regulates p27 levels through Skp2-mediated p27 degradation.

### SIRT2 decreases Skp2 levels through induction of Skp2 deacetylation

**Figure 6:**
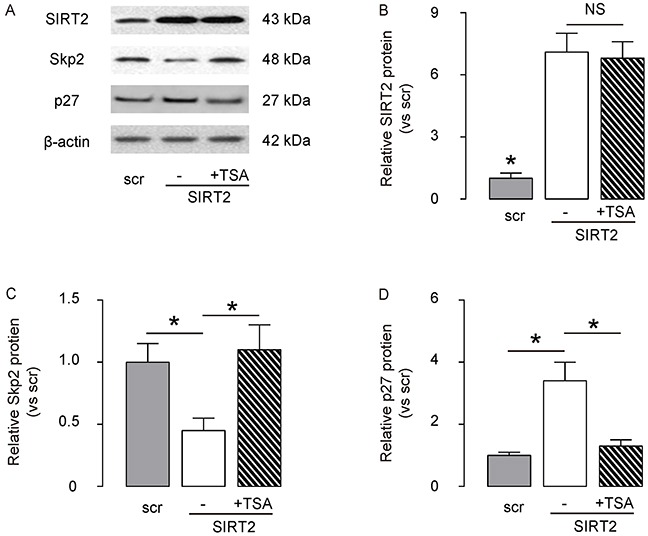
SIRT2 decreases Skp2 levels through induction of Skp2 deacetylation The SIRT2-overexpressing A549 cells were exposed with TSA, an effective inhibitor for the class I and II HDACs, including SIRT2. **A.** The levels of SIRT2, Skp2 and p27 in SIRT2-depleted A549 cells with/with TSA were analyzed by Western blot, shown by representative Western blot. **B–D.** Quantification for SIRT2 (B), Sep2 (C) and p27 (D) protein in SIRT2-modified A549 cells. *p<0.05. NS: non-significant. N=5.

Then, we examined how SIRT2 regulated Skp2 levels in NSCLC cells. Based on literature, Skp2 may induce the Skp2 deacetylation in some types of cells, and deacetylated Skp2 is not stable and undergoes degradation. Since we have shown that SIRT2 inhibits Skp2 in NSCLC cells, we thus evaluated whether SIRT2 may regulate Skp2 levels through induction of Skp2 deacetylation. We exposed SIRT2-overexpressing A549 cells with TSA, an effective inhibitor for the class I and II mammalian histone deacetylase (HDAC) families of enzymes, including SIRT2. We found that MG132 did not alter the levels of SIRT2 in SIRT2-overexpressing A549 cells, by representative Western blot (Figure 6A) and by quantification (Figure 6B), but abolished the effects of SIRT2 overexpression on the suppression of Skp2, by representative Western blot (Figure 6A) and by quantification (Figure 6C). Moreover, the changes in Skp2 levels resulted in adaption of p27 levels, by representative Western blot (Figure 6A) and by quantification (Figure 6D). Hence, our data suggest that SIRT2 decreases Skp2 levels through induction of Skp2 deacetylation.

### SIRT2 and Skp2 are associated in NSCLC cells

**Figure 7:**
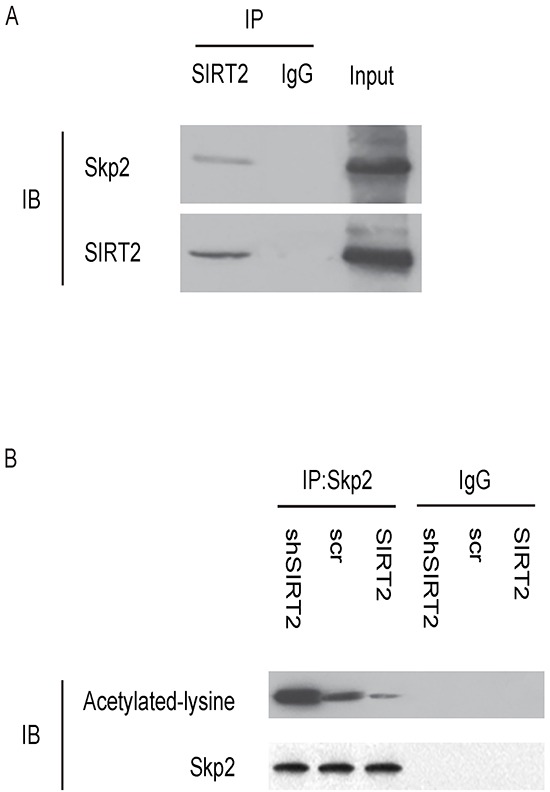
SIRT2 and Skp2 are associated in NSCLC cells **A.** Immunoprecipitation analyses showing that SIRT2 was co-immunoprecipitated with Skp2 in A549 cells. **B.** Using an anti-acetylated-lysine antibody in immunoprecipitation, we found that the acetylation level of Skp2 significantly increased in SIRT2-depleted A549 cells, and significantly decreased in SIRT2-overexpressing cells.

**Figure 8:**
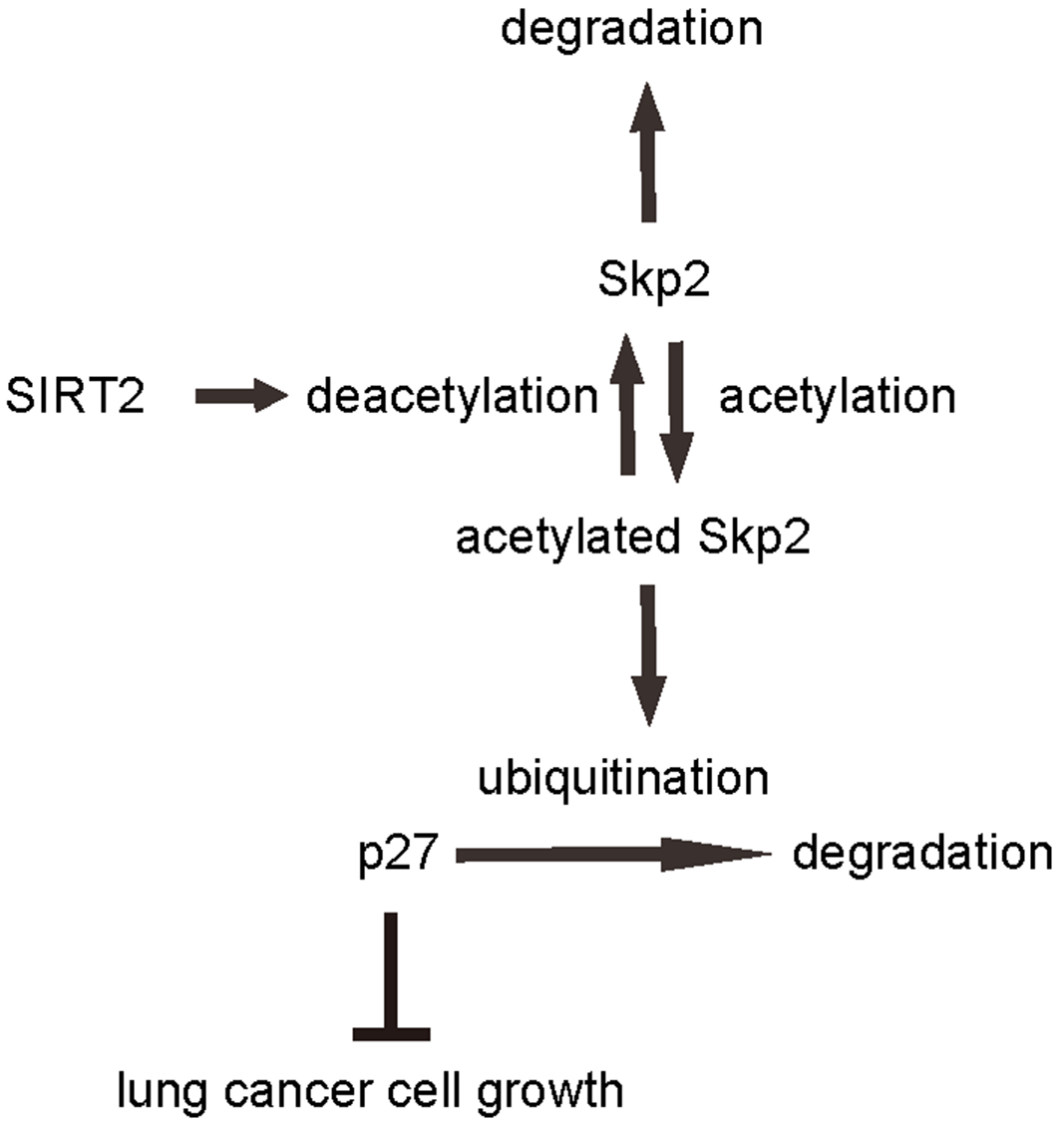
Schematic of the model Illustration of a novel role of SIRT2/Skp2/p27 in control of NSCLC cell growth. SIRT2 regulates Skp2 levels through induction of protein deacetylation and degradation, while Skp2 regulates of p27 through induction of ubiquitination-associated protein degradation.

To confirm the above findings and further demonstrate a regulatory relationship between SIRT2 and Skp2 in NSCLC cells, we performed immunoprecipitation analyses. First, SIRT2 was co-immunoprecipitated with Skp2 in A549 cells (Figure 7A). Using an anti-acetylated-lysine antibody, we found that the acetylation level of Skp2 significantly increased in SIRT2-depleted A549 cells, and significantly decreased in SIRT2-overexpressing cells (Figure 7B). Together, these data revealed a novel role of SIRT2 in regulating NSCLC growth through deacetylation of Skp2, which in turn altered the level of p27 through ubiquitination-associated protein degradation (Figure 8).

## DISCUSSION

Identification of key genes involved in the carcinogenesis of lung cancer may provide new insight into development of novel therapeutic strategies. Protein stability, function and degradation are regulated by various mechanisms, including acetylation, deacetylation, ubiquitination and de-ubiquitination, the aberrant alterations of which have been acknowledged to play a critical role in tumor initiation, progression, growth and metastases.

Degradation of the cyclin-dependent kinase (CDK) inhibitor p27 is required for the cellular transition from quiescence to the proliferative state in many cell types. The ubiquitination and subsequent degradation of p27 have been shown to require its phosphorylation by cyclin-CDK complexes. A recent study by Carrano et al. [23] showed that the Skp2 specifically recognized p27 in a phosphorylation-dependent manner and induced ubiquitination and subsequently degradation of p27 to allow the cells to proliferate. Although this paper used HeLa cells and fibroblasts to draw the conclusion, follow-up studies have shown that this molecular machinery also works in many other cell types [21-23].

SIRT2 is a histone deacetylase that is ubiquitously expressed in normal tissues, but get lost in many primary human tumors and tumor cell lines [35-38]. Moreover, re-expression of SIRT2 has been found to inhibit tumor cell growth in vitro [35-38]. Ying et al. reported that SIRT2 levels were significantly reduced in lung cancer, and SIRT2 appeared to increase cellular autophagy activity through unknown mechanism [39]. Chen et al. showed that SIRT2 contributed to cell motility and invasiveness of hepatocellular carcinoma (HCC). SIRT2 was up-regulated in HCC cell lines and in a subset of human HCC tissues. Up-regulation of SIRT2 in primary HCC tumors were significantly correlated with the presence of microscopic vascular invasion, a more advanced tumor stage, and shorter overall survival [40]. The authors also showed that short hairpin RNA-mediated suppression of SIRT2 expression in HCC cell lines suppressed cancer cell motility and invasiveness, by induction of regression of epithelial-mesenchymal transition. Most importantly, they also showed that SIRT2 regulated the deacetylation and activation of protein kinase B [40]. Recently, we reported that SIRT2 level was down-regulated in the NSCLC cells. Overexpression of SIRT2 in lung cancer cell lines modulated cell proliferation, apoptosis and cell cycle, and enhanced the sensitivity to Cisplatin-induced cytotoxicity. Moreover, overexpression of SIRT2 increased cellular p27 [20].

Both SIRT2 and Skp2 altered p27 levels, while Skp2 is well-known to be regulated by acetylation and deacetylation [41], and SIRT2 is a potent histone deacetylase. Thus, we asked whether there may be a regulatory relationship between SIRT2 and Skp2, specifically in NSCLC cells. This question has not been answered previously and was addressed in the current study.

Here, we found that NSCLC specimens had significant lower levels of SIRT2 and higher levels of Skp2, compared to NT. The levels of SIRT2 and Skp2 were inversely correlated. These clinical findings are bases for our in vitro mechanistic analyses and a demonstration of the clinic relevance of this study.

In vitro, the levels of SIRT2 and Skp2 in NSCLC cell lines are consistent with primary NSCLC. Modification of SIRT2 levels directly regulated Skp2 levels in NSCLC cells. Moreover, inhibition of deacetylase activity of SIRT2 in SIRT2-overexpressing cells abolished the suppressive effects of SIRT2 on Skp2, suggesting that SIRT2 indeed regulates Skp2 through deacetylation-mediated induction of instability of the protein to cause Skp2 degradation. On the other hand, the changes in p27 levels by SIRT2-regulated Skp2 could be abolished by proteasome inhibitor, suggesting that the regulatory axis Skp2/p27 also functions in NSCLC cells.

Notable, a recent study showed that the mechanism of cell senescence avoidance during contact inhibition is associated with induction of p27 and CDK inhibitors, and inhibition of the mTOR pathway [42]. The results from this study may partially explain our findings here, in which SIRT2/Skp2/p27 controls NSCLC cell growth, in which SIRT2 appears to be a tumor suppressor and Skp2 appears to be an oncogene. SIRT2 regulates Skp2 levels through induction of protein deacetylation and degradation, while Skp2 regulates of p27 through induction of ubiquitination-associated protein degradation. Manipulation of any part in this pathway may affect NSCLC cell growth, which may provide innovative strategies for treating lung cancer.

## MATERIALS AND METHODS

### Patient specimens

Resected cancer specimens from 28 NSCLC patients were obtained together with the paired tumor-adjacent normal lung tissues (NT) from 2009 to 2013 at the Shanghai Lung Tumor Clinical Medical Center of China. All patients provided signed, informed consent for their tissues to be used for scientific research. Ethical approval for the current study was obtained from the Shanghai Lung Tumor Clinical Medical Center. All diagnoses were based on pathological and/or cytological evidence. The histological features of the specimens were evaluated by 2 experienced pathologists independently, according to the World Health Organization classification criteria. Tissues were obtained prior to chemotherapy and radiotherapy and were immediately frozen and stored at −70°C prior to Western blot analyses.

### Lung cancer cell lines

Six human lung cancer cell lines were included in the current study, and were all purchased from American Type Culture Collection (ATCC, Rockville, MD, USA). A549 cell line was first developed in 1972 by Dr. Giard through the removal and culturing of cancerous lung tissue in the explanted tumor a of 58-year-old caucasian male [43]. Calu-6 was an anaplastic lung carcinoma cell line, Calu-3 is a lung adenocarcinoma cell line, RPMI-2650 is a lung anaplastic squamous cell carcinoma cell line, A-427 is a pulmonary carcinoma cell line, NCI-H292 is a lung mucoepidermoid carcinoma cell line. All these cell lines were cultured in RPMI 1640 medium supplemented with 15% heat-inactivated fetal bovine serum (FBS; Sigma-Aldrich, St Louis, MO, USA), 100U/ml penicillin, and 100μg/ml streptomycin (Invitrogen, Carlsbad, CA, USA) in a humidified atmosphere of 5% CO_2_ at 37°C.

### Reagents

Trichostatin A (TSA, Sigma-Aldrich) is an organic compound that serves as an antifungal antibiotic which selectively inhibits the class I and II mammalian histone deacetylase (HDAC) families of enzymes, including SIRT2. TSA was used in 1μmol/l in culture to inhibit SIRT2 activities on deacetylation. MG132 (Sigma-Aldrich) is a specific, potent, reversible and cell-permeable proteasome inhibitor to reduce the cellular degradation of ubiquitin-conjugated proteins. MG132 was used in 5μmol/l in culture to inhibit p27 ubiquitination-associated degradation.

### Transfections

SIRT2 and p27 transgenes, short-hairpin interfering RNA for SIRT2 (shSIRT2; sequence: 5′-GAGGCCAUCU UUGAGAUCAdTdT-3′) and control scrambled sequence (scr) were obtained from Origene (Beijing, China). These plasmids were used to modify either SIRT2 levels or p27 levels in A549 cells through transfection at a concentration of 50nmol/l using Lipofectamine 2000 (Invitrogen), according to the manufacturer's instruction. The transfection was analyzed one day after, showing more than 95% efficiency.

### MTT assay

For assay of cell viability, cells were seeded into 24 well-plate at 10000 cells per well and subjected to a Cell Proliferation Kit (MTT, Roche, Indianapolis, IN, USA), according to the instruction of the manufacturer. The MTT assay is a colorimetric assay for assessing viable cell number, taking advantage that NADPH-dependent cellular oxidoreductase enzymes in viable cells reduce the tetrazolium dye 3-(4,5-dimethylthiazol-2-yl)-2,5-diphenyltetrazolium bromide (MTT) to its insoluble formazan in purple readily being quantified by absorbance value (OD) at 570 nm. Experiments were performed 5 times.

### Proliferation assay

For analysis of apoptosis, cultured cells were dissociated and re-suspended at a density of 106 cells/ml in PBS. After staining with FITC-conjugated BrdU antibody (FITC BrdU Flow Kit, Becton-Dickinson Biosciences), cells were analyzed using FACScan flow cytometer (Becton-Dickinson Biosciences) equipped with Cell Quest software (Becton-Dickinson Biosciences) for determination of FITC+ S-phase proliferating cells.

### Immunoprecipitation and western blot

Cells were lysed with lysis buffer (50mmol/l Tris, 50mmol/l KCl, 20mmol/l NaF, 1mmol/l Na_3_VO_4_, 10mmol/l EDTA, 1% NP-40, 10mmol/l nicotinamide, 1mmol/l TSA (Trichostatin A), 1mmol/l phenylmethanesulfonyl fluoride, 5 mg/ml leupeptin, pH 8.0). For immunoprecipitation, cell lysates were incubated with 20 ml anti-Flag agarose beads (Sigma-Aldrich) at 4°C with overnight shaking. After being washed five times with lysis buffer, the precipitated proteins were resolved by sodium dodecyl sulfate–polyacrylamide gel electrophoresis followed by immunoblot analysis. For endogenous protein-protein interaction, the lung cancer cells with or without SIRT2 knockdown were lysed, and followed by incubation with anti-Skp2 monoclonal antibody or control immunoglobulin G at 4°C overnight. The immunocomplexes were then precipitated with BioMag Goat antimouse immunoglobulin G magnetic beads (Polysciences, Warrington, PA, USA) and followed by immunoblot analysis.

For Western blot, proteins were separated on SDS-polyacrylamide gels. The separated proteins were then transferred to a PVDF membrane. The membrane blots were first probed with a primary antibody. After incubation with horseradish peroxidase-conjugated second antibody, autoradiograms were prepared using the enhanced chemiluminescent system to visualize the protein antigen. The signals were recorded using X-ray film. Primary antibodies were rabbit anti-SIRT2, anti-Skp2, anti-p27 and anti-β-actin (Cell Signaling, San Jose, CA, USA). Secondary antibody is HRP-conjugated anti-rabbit (Jackson ImmunoResearch Labs, West Grove, PA, USA). β-actin was used as a protein loading control. The protein levels were first normalized to β-actin, and then normalized to the experimental control.

### Quantitative real-time PCR (RT-qPCR)

MiRNA and total RNA were extracted from lung specimen or from the cultured cells with miRNeasy mini kit or RNeasy kit (Qiagen, Hilden, Germany), respectively. For cDNA synthesis, complementary DNA (cDNA) was randomly primed from 2μg of total RNA using the Omniscript reverse transcription kit (Qiagen). RT-qPCR was subsequently performed in triplicate with a 1:4 dilution of cDNA using the Quantitect SyBr green PCR system (Qiagen) on a Rotorgene 6000 series PCR machine. All primers were purchased from Qiagen. Data were collected and analyzed using 2-ΔΔCt method. Values of genes were first normalized against β-actin, and then compared to controls.

### Statistical analysis

All statistical analyses were carried out using the SPSS 18.0 statistical software package. All data were statistically analyzed using one-way ANOVA with a Bonferroni correction, followed by Fisher's exact test. Bivariate correlations were calculated by Spearman's rank correlation coefficients. Kaplan-Meier curves were sued to analyze the patient survival by SIRT2 levels. All values are depicted as mean ± standard deviation and are considered significant if p < 0.05.
